# Homocysteine and Digestive Tract Cancer Risk: A Dose-Response Meta-Analysis

**DOI:** 10.1155/2018/3720684

**Published:** 2018-12-18

**Authors:** Jun Xu, Xin Zhao, Shanwen Sun, Peng Ni, Chujun Li, Anjing Ren, Wei Wang, Lingjun Zhu

**Affiliations:** ^1^Department of Oncology, The First Affiliated Hospital of Nanjing Medical University, Nanjing, China; ^2^Department of Respiratory and Critical Care Medicine, The First Affiliated Hospital of Nanjing Medical University, Nanjing, China; ^3^Department of Plastic and Burns Surgery, The First Affiliated Hospital of Nanjing Medical University, Nanjing, China; ^4^Department of Thoracic Surgery, The First Affiliated Hospital of Nanjing Medical University, Nanjing, China

## Abstract

**Background:**

Homocysteine, a key component in one-carbon metabolism, is of great importance in remethylation. Many epidemiologic studies have assessed the association between homocysteine and risk of digestive tract cancer, but the results are inconsistent.

**Objective:**

The objective of our meta-analysis is to assess the association between homocysteine and digestive tract cancer risk.

**Methods:**

Comprehensive searches were performed on the PubMed, Embase, Cochrane, and Web of Science databases up to September 25, 2018, to identify relevant studies. Thirteen studies were included in the meta-analysis. Odds ratios (ORs) and their corresponding 95% confidence intervals (95% CIs) were used to estimate the strength of the relationship between homocysteine and the risk of digestive tract cancer.

**Results:**

The pooled OR of digestive tract cancer risk for patients with the highest categories of blood homocysteine levels versus the lowest categories was 1.27 (95% CI, 1.15, 1.39) with no significant heterogeneity observed (*P *= 0.798,* I*^*2*^ = 0.0%). Moreover, the dose-response analysis revealed that each 5*μ*mol/L increase in homocysteine increased the incidence of digestive tract cancer by 7%.

**Conclusion:**

Generally, our results indicated that elevated homocysteine was associated with higher risk of digestive tract cancer. That is, homocysteine concentration may be a potential biomarker for occurrence of digestive tract cancer.

## 1. Introduction

Digestive tract cancers, which refer to malignant diseases of the gastrointestinal tract and mainly including esophageal, gastric, and colorectal cancers, are still major public health burdens. According to Globocan 2012 estimates, the standardized incidences of colorectal cancer, stomach cancer, and esophageal cancer were the 3rd, 5^th^, and 8th most common, respectively, of all tumors, making up 19.7 percent of new cancer cases. Gastric cancer, ranked as the third leading cause of cancer death, results in 723,000 deaths in both sexes annually all over the world. In total, digestive tract cancer constitutes 22.2 percent of the mortality due to cancer, leading to 1,817,000 cases of cancer death every year [1].

Previous studies have found that DNA methylation, which varies in the development of many cancer types, has a link to cancer [2]. Hypermethylation or hypomethylation, in which one-carbon metabolites play an important role, can be observed in a wide variety of malignancies, including gastric, esophageal, and colorectal cancers [3, 4]. If substances that reflect cellular methylation status can be found, these cancers could be found in the early stages and millions of people could cheat death and live longer lives. One-carbon metabolic reactions mainly include two biological processes: the synthesis of purines and pyrimidines, which are necessary for DNA replication and repair, and the synthesis of S-adenosylmethionine (SAM), a methyl group donor for a number of methylation reactions, including DNA, RNA, and protein methylation. Homocysteine, as the intersection of the methylation, remethylation and transsulfuration pathways, is an important component in the one-carbon metabolism. S-adenosylhomocysteine produces homocysteine catalyzed by S-adenosylhomocysteine hydrolase, which is a reversible reaction. Therefore, homocysteine is intrinsically linked to cellular methylation status. Methionine synthase produces methionine by remethylating homocysteine, using the methyl group from betaine, or from 5-methyltetrahydrofolate, derived from the metabolism of 5,10-methylene tetrahydrofolate by 5,10-methylene tetrahydrofolate reductase. In the transsulfuration pathway, homocysteine produces cystathionine, the precursor for cysteine biosynthesis, catalyzed by vitamin B6-dependent cystathionine *β*-synthase [5]. The pathway which involves the transamination of homocysteine to form methionine can be affected by folic acid and vitamin B12 [6]. Deficiencies of folate and vitamin B12 can first block the reaction of homocysteine remethylation and, second, block homocysteine catabolism because of the reduced synthesis of SAM [7]. The relationship between folic acid and digestive cancer has been discussed by various studies, but epidemiological data are inconsistent [8–12]. Additionally, many studies have focused on the influence of the B vitamins on the risk of digestive tract cancer [13]. Studies found that total homocysteine concentration can represent one-carbon metabolism related nutrients, especially folic acid and B vitamins [14].

Homocysteine has been widely studied in terms of cardiovascular disease and has been recognized as a potential risk factor for stroke, coronary vascular disease, ischemic heart disease, and other vascular occlusive diseases [15, 16]. Besides, the effect of homocysteine on cancer has also been discussed. So far, several epidemiologic studies have also assessed the relation of homocysteine to the incidence of digestive tract cancer, but the results are inconsistent. For example, data from Lina Wang et al. [17] and Miller et al. [18] showed that an increased occurrence of digestive tract cancer was observed in people with higher levels of homocysteine than in those with lower levels. However, some studies on this topic showed a nonsignificant increase in the risk of digestive tract cancer in populations with the highest levels of homocysteine [19, 20]. One meta-analysis performed by Collin et al. demonstrated the relationship between blood total homocysteine and prostate cancer [21]. Another meta-analysis written by Xu et al. found that higher blood homocysteine levels increased gastric cancer risk [22].

Based on the central role of homocysteine in carbon metabolism, its predictive role in cardiovascular disease, and the inconsistency of existing epidemiologic studies and inadequate statistical power in many primary studies, we chose to study the role of homocysteine in digestive tract cancers. The meta-analysis was conducted to assess the evidence from the literature on the relationship between blood levels of homocysteine and the risk of digestive tract cancers and to further to evaluate the dose-response relationship. We hope that we can provide a basis for the use of homocysteine as a tumor marker of digestive tract cancers in the future.

## 2. Materials and Methods

### 2.1. Search Strategy

We conducted a comprehensive, computerized literature search of the PubMed, Embase, Cochrane, and Web of Science databases for relevant epidemiologic studies with the aim of investigating the connection between homocysteine and digestive tract cancers published on September 25, 2018. The key words used were as follows: (1) gastrointestinal neoplasms, colorectal neoplasms, colorectal carcinoma, colorectal cancer, colonic neoplasms, colonic carcinoma, colonic cancer, colon neoplasms, colon carcinoma, colon cancer, rectal neoplasms, rectal carcinoma, rectal cancer, rectum neoplasms, rectum cancer, rectum carcinoma, esophageal neoplasms, esophageal cancer, esophageal tumor, esophageal carcinoma, gastric neoplasms, gastric cancer, gastric tumor, gastric carcinoma, stomach neoplasms, stomach cancer, stomach tumor, and stomach carcinoma; (2) homocysteine, 2-amino-4-mercaptobutyric acid, hcy, thcy, and hyperhomocysteinemia. In addition, references from the relevant original reports were also scrutinized and hand-searched. No restrictions were imposed. We also made efforts to contact the authors of the primary studies for additional information. Our meta-analysis did not include unpublished reports.

### 2.2. Study Selection

Studies were recognized as eligible for our analysis if they met the following criteria: (1) the study design was a case-control or cohort study and investigated the association between homocysteine and digestive tract cancer incidence; (2) articles were written in English; (3) two comparison groups (cancer group vs. control group) were included in the study; (4) the outcome of this review, the incidence risk data of univariate and/or multivariate analyses presented as OR, had a 95% CI when comparing the highest levels of homocysteine with the lowest levels.

The exclusion criteria were as follows: (1) the articles only composed of an overview and summary (without data); (2) animal study; (3) the same population in another study; and (4) studies with incomplete data.

### 2.3. Data Extraction

The two authors independently extracted all the data according to the selection criteria and reached a consensus on all projects. If there was a disagreement, another author would reevaluate these articles. All data was extracted using a standardized data-collection form. Study features were recorded as follows: (1) name of first author and publication year; (2) cancer type; (3) the location of the study conducted and study design; (4) the number of cases and controls; (5) method of measurement; (6) gender and age of cases; (7) source of controls; (8) ORs from the most fully adjusted model for the highest homocysteine compared with the lowest homocysteine and their corresponding 95% CIs; and (9) confounders adjusted for in multivariate analysis. Homocysteine was expressed uniformly as *μ*mol/L.

We used a scale called the Newcastle-Ottawa-Scale (NOS) to assess the quality of eligible studies. According to the NOS, studies were evaluated according to 3 aspects, including selection, comparability, and measurement of exposure for case-control studies or outcome for cohort studies. The NOS scale ranges from zero to nine stars. A study with ≥ 6 stars would be regarded as a high-quality study [31].

### 2.4. Statistical Analysis

Odds ratios and their corresponding 95% confidence intervals were used to measure the strength of the correlation between homocysteine and the risk of digestive cancer. The Q-test and the* I*^*2*^ (inconsistency index) statistic were calculated to quantify the proportion of total variation attributable to heterogeneity between eligible studies [32]. Generally,* P *< 0.05 corresponded to heterogeneity across eligible studies, and a random-effects model (DerSimonian and Laird method) was appropriate to compute the summary risk estimates. Otherwise, the fixed-effects model (the Mantel-Haenszel method) was preferred. Moreover,* I*^*2*^ values >50% represented strong heterogeneity between studies and* I*^*2*^ values < 25% suggested no significant heterogeneity [33]. We also conducted an influence analysis in which one study was omitted at a time and the rest were analyzed to investigate the effect of a single study on the overall result. The evidence of potential publication bias was assessed using Begg's funnel plot and Egger's linear regression test and* P *< 0.05 represented significant publication bias [34, 35]. All statistical analyses were performed using Stata software (version 12.0, USA).

To estimate the trend in correlated OR with the elevated concentration of homocysteine, we conducted a dose-response analysis using the method recommended by Greenland and Longnecker [36] and the publicly available Stata code written by Orsini et al. [37]. At least 3 quantitative exposure categories of homocysteine with corresponding median or mean values of homocysteine for each category, distributions of cases and noncases, and ORs and its corresponding 95% CIs were necessary for the analysis. Supposing that studies did not report median or mean values of concentration, we used the midpoint of each category. If the highest or lowest category was open-ended, the midpoint of each category was estimated by assuming that the width of the category was the same as the next adjacent category. The dose-response results are presented for a 5*μ*mol/L increment.

## 3. Results

### 3.1. Characteristics of the Studies

**Figure 1:**
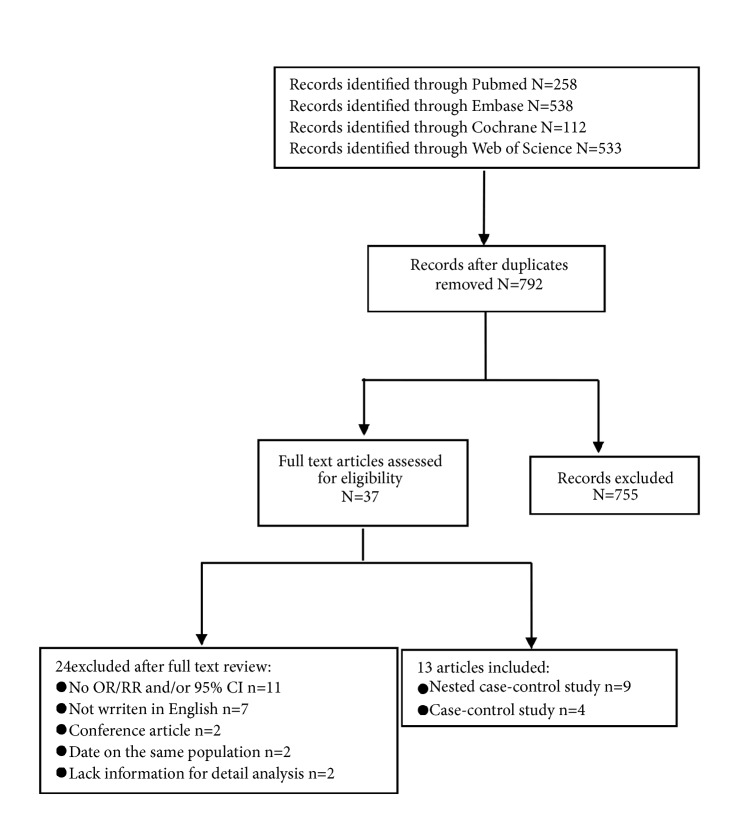
Flow diagram of the study identification and selection.

By the search strategy, a total of 792 citations were obtained from the PubMed, Embase, Cochrane, and Web of Science databases, of which 37 eligible studies were considered of potential value; then, we retrieved the full text for further evaluation. According to inclusion criteria, 24 of the 37 articles were subsequently excluded from the meta-analysis (11 did not report OR and/or 95% CI, 7 were not written in English, 2 were conference articles, 2 reported data on the same population, and 2 lacked information for detailed analysis). Finally, 13 articles [8, 17–20, 23–30] (4 case-control and 9 nested case-control studies) met our inclusion criteria (Figure 1). Details of these studies are presented in Table 1.

### 3.2. Overall and Subgroup Results

**Figure 2:**
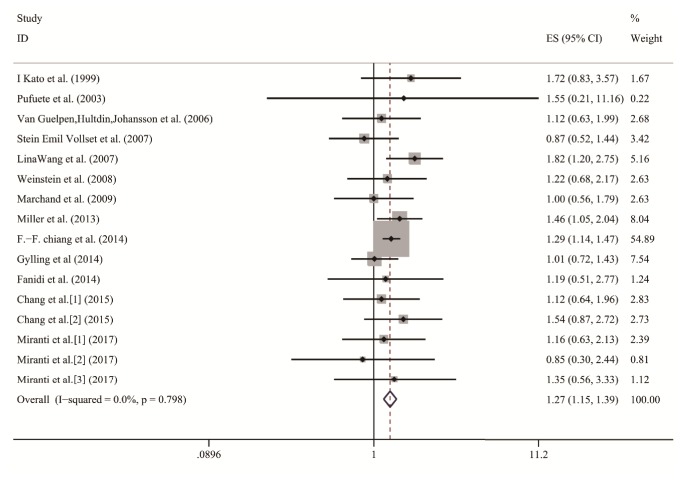
Forest plot of highest versus lowest categories of homocysteine on digestive cancer risk. The squares and horizontal lines correspond to the study-specific ORs and 95% CI. The area of the squares reflects the weight. The diamond represents the summary OR and 95% CI. ORs, Odds ratios; CI, confidence interval.

The multivariable-adjusted ORs for each study and all studies combined for the highest compared with the lowest categories of homocysteine level are shown in Figure 2. The pooled OR of digestive cancer for the highest compared with the lowest categories of blood homocysteine level was 1.27 (95% CI, 1.15, 1.39) with no significant heterogeneity observed (*P *= 0.798,* I*^*2*^ = 0.0%). Thus, we utilized fixed-effects models to analyze the association. Significantly increased incidence of digestive tract cancer was observed in patients with high homocysteine levels compared with those with low levels, from the data.

Firstly, in the subgroup analysis of study design, the OR was 1.16 (95% CI, 0.99-1.37) for nested case-control studies and 1.33 (95% CI, 1.18-1.49) for case-control studies. We further analyzed the results by study location: studies conducted in Asia (OR = 1.33; 95% CI: 1.18, 1.49) and America (OR = 1.38; 95% CI: 1.05, 1.80) indicated that higher homocysteine was associated with the increased occurrence of digestive cancer in people living in Asia and America. However, we did not see this effect in Europeans (OR = 1.06; 95% CI: 0.87, 1.30). Additionally, the subgroup analysis of cancer type suggested that colorectal cancer risk was positively associated with homocysteine levels (OR = 1.27; 95% CI: 1.14, 1.41), while the relationship became weaker in esophageal cancer (OR = 1.31; 95% CI: 0.89, 1.93) and gastric cancer (OR = 1.27; 95% CI: 0.98, 1.64). Of the 13 articles, 3 articles measured homocysteine in serum (OR = 1.26; 95% CI: 0.91, 1.74), and 10 articles measured homocysteine in plasma (OR = 1.27; 95% CI: 1.15, 1.40). Additionally, in the subgroup analysis of method of measurement, the positive association was still significant in studies using HPLC (OR = 1.29; 95% CI: 1.6, 1.44), while the association was not significant in studies using GC-MS and FPIA. Details can be seen in Table 2.

### 3.3. Dose-Response Analysis

**Figure 3:**
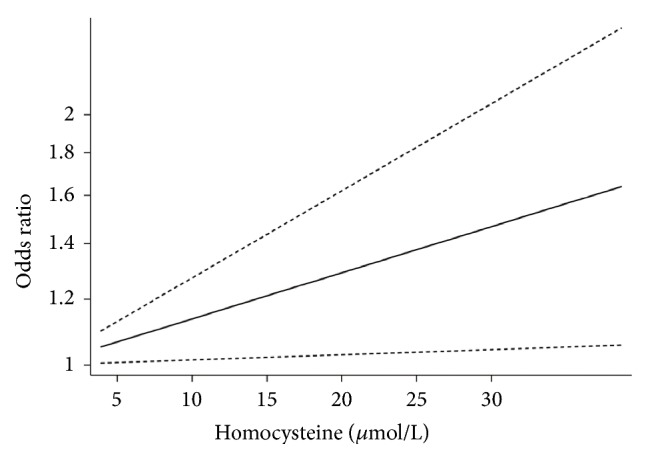
Dose-response analyses of the linear association between homocysteine and the risk of digestive cancer.

Only 5 articles [17, 20, 25, 28, 29] composed of 6 comparisons from all 13 studies were eligible for exploring the dose-response relationship between the concentration of homocysteine and digestive cancer risk. The summary OR per 5*μ*mol/L increase in homocysteine was 1.07 (95% CI, 1.01 to 1.19) (Figure 3) which revealed that each 5*μ*mol/L increase in homocysteine was associated with a 7% higher risk of digestive cancer occurrence.

### 3.4. Influence Analysis

To assess the stability of the results, we performed an influence analysis in which one study was omitted at a time each term and we calculated the pooled ORs for the rest of the studies. The analysis showed that the result was mostly affected by the study of F.-F. Chiang et al. (Figure 5). After excluding the study, the pooled OR was 1.24 (95% CI, 1.08-1.43), suggesting there were no changes in the direction of the effect which indicated good stability of our meta-analysis.

### 3.5. Publication Bias Analysis

**Figure 4:**
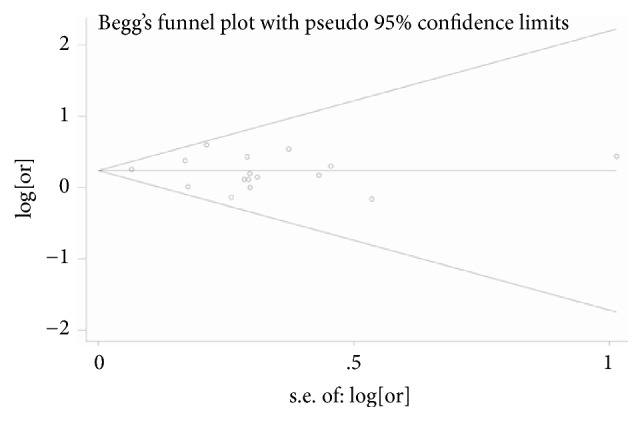
Begg's funnel plot for publication bias test of the relationship between homocysteine and digestive cancer risk.

**Figure 5:**
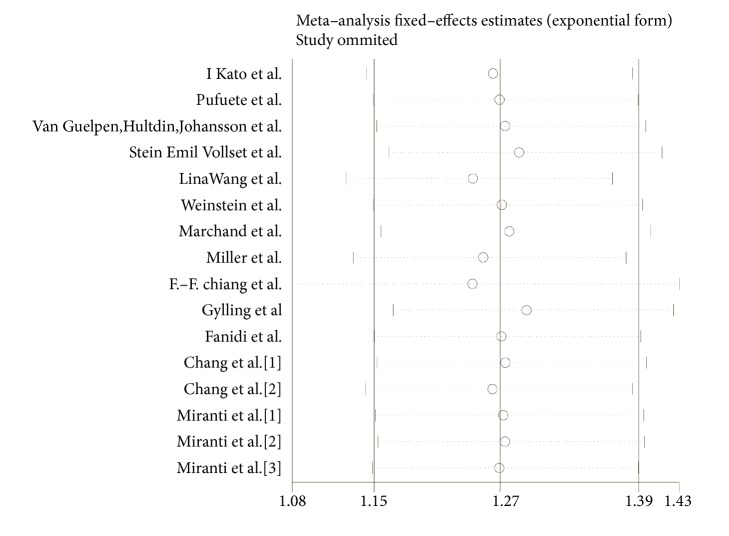
Influence analysis of the pooled relative risk coefficients on the relationship between homocysteine and digestive cancer risk. The two ends of the dotted lines represent the 95% CI.

Begg's funnel plot and Egger's test were conducted to evaluate the publication bias of the studies included. The shape of the funnel plots seemed symmetrical (Figure 4) and the* P* value of Egger's test was 0.59 (*P *> 0.05), which indicated that there was no existence of publication bias.

## 4. Discussion

On the basis of the pooled OR of our meta-analysis, people with higher homocysteine were more likely to suffer from digestive tract cancer. According to the influence analysis, we can see that after removing the study of F.-F. Chiang et al., the pooled OR was changed most greatly, but the trend did not change, which indicated the stability of our result. The rest of the article included in our analysis classified the level of homocysteine by three or more categories, but the study of F.-F. Chiang et al. divided homocysteine levels into two categories (higher and lower), which may be the reason why it had significant influence on the pooled results. Moreover, the dose-response analysis further demonstrated the positive effect of homocysteine on digestive tract cancer: each 5*μ*mol/L increase in concentration of homocysteine enhanced the chance of developing digestive cancer by 7%.

When stratified by study design, the positive association between homocysteine and digestive cancer risk was only seen in case-control studies and was weaker in nested case-control studies. Such results can be explained because it is difficult to distinguish the chronological sequence of exposure and occurrence of diseases because of the retrospective nature of case-control studies. Additionally, according to the subgroup analysis of location, elevated homocysteine levels seemed to have more predictive value for digestive cancer in people living in Asia and America. Moreover, in the separate analysis of cancer type, the positive association between homocysteine and digestive cancer risk was weaker in esophageal cancer and gastric cancer. The discrepancy in results could be attributed to the fact that there are limited studies on the relationship between homocysteine, the esophagus and gastric cancer risk, which affected the stability summary effects. Thus, more studies are needed to confirm our conclusion.

As we know, runaway gene expression and the unbalanced genome integrity whose major epigenetic mechanism is DNA methylation are considered causes of oncogenesis [38]. DNA methylation is the most relevant epigenetic feature in the clinic, in which a methyl group is covalently added to the cytosine of the CpG dinucleotide. Extensive hypomethylation and hypermethylation in normally unmethylated gene promoter CpG islands are considered to be the most commonly studied epigenetic alterations in cancer and affect the stability and function of genes. When the promoter region of the tumor suppressor gene is hypermethylated, its expression can be inhibited, resulting in genetic mutation and increased cell proliferation. Additionally, studies have demonstrated that hypermethylation could be in the promoter region of mismatch repair gene hMLH1 in patients with sporadic colorectal cancer with microsatellite instability, which can be used as a basis for treatment and prognosis [39, 40]. All of these indicate that methylation is closely related to tumorigenesis and that homocysteine can be used as a marker of methylation status in vivo.

However, the mechanisms by which elevated homocysteine enhances digestive tract carcinogenesis have not yet been clearly elucidated. Homocysteine is a non-protein–forming sulfur amino acid whose metabolism is of great importance in remethylation, in which SAM plays the part of a universal methyl donor to various acceptors [41]. A byproduct of the remethylation reaction is S-adenosylhomocysteine (SAH), which is hydrolyzed and subsequently regenerates homocysteine. It should be noted that this reaction is reversible with equilibrium dynamics that have a preference for SAH synthesis to hydrolysis. The reason why this reaction actually proceeds in the hydrolytic direction is that the product can be removed efficiently [42]. If the homocysteine cannot be removed effectively, the SAH will accumulate which has a negative influence on the methyltransferase reaction [43]. Increasing concentrations of homocysteine were found to be linked to increased concentrations of SAH. With the increase of SAH levels, intracellular lymphocyte SAH levels increase, which results in hypomethylation of lymphocyte DNA [44]. All the above suggests that elevation of homocysteine levels may have a negative and indirect influence on the cellular methylation reactions that were found to have a close link to the pathogenesis of cancer.

As far as we know, this meta-analysis is the first to explore the relationship between homocysteine and digestive cancer risk by the method of dose-response analysis. Additionally, there was no significant heterogeneity or publication bias observed in our analysis, which suggested that the articles included were compatible for our meta-analysis despite including a variety of studies concerning different populations, living environments, family history, habits, and customs. However, we must acknowledge the limitations of the meta-analysis. First, unpublished studies were not included in our meta-analysis, which can result in publication bias although the statistical data did not reflect this. Second, only studies published in English met our inclusion criterion, so meaningful data may have been ignored. Additionally, only 13 comparisons were contained in the meta-analysis, which might, to a certain extent, have affected the reliability of the results.

## 5. Conclusion

In conclusion, our meta-analysis suggested that, with higher concentration of homocysteine, the incidence of digestive tract cancer increased despite the limitations of our meta-analysis. Further studies with larger sample size and more integral data are necessary to confirm our finding.

## Figures and Tables

**Table 1:** Main features of the included studies for the association between homocysteine and digestive tract cancer.

First author, (year of publication)	Cancer type	Country; study design	Source of control	Case/ Control	Method of measurement /Type of homocysteine)	Gender	Median or mean age of (y)	Contrast (highest *vs.* lowest) (*µ*mol/L)	Adjusted OR (95% CI) (highest vs. lowest)	Adjustment	Quality score
I Kato et al. (1999) [23]	colorectal cancer	US; Nested case-control	PB	105/ 523	HPLC /Serum	F	66.2	Quartile ≥12.21 *vs* ≤7.9	1.72 (0.83-3.57)	Adjusted for family history of colorectal cancer, beer intake, prior occult blood testing and number of hours spent in sport activities in their early 30s.	8
Pufuete et al. (2003) [24]	colorectal cancer	England; case-control	HB	28/ 76	FPIA /Plasma	M/F	58	Top third *vs* bottom third	1.55 (0.21-11.16)	Adjusted for sex, age, body mass index, smoking, alcohol intake, MTHFR, MS, and CBS genotypes.	7
Van Guelpen, Hultdin, Johansson et al. (2006) [19]	colorectal cancer	Sweden; Nested case-control	PB	226/ 437	FPIA /Plasma	M/F	59.8	Quintile Q5 *vs* Q1	1.12 (0.63-1.99)	Adjusted for body mass index, current smoking, recreational and occupational physical activity, and alcohol intake (alcohol data were available for approximately two thirds of subjects).	8
Stein Emil Vollset et al. (2007) [8]	gastric cancer	EPIC; Nested case-control	PB	245/ 631	GC-MS/ Plasma	M/F	62.2	Q5 *vs* Q1	0.87 (0.52-1.44)	Adjusted for Hp status, smoking, and energy in conditional logistic model with stratification on matched sets.	8
Lina Wang et al. (2007) [17]	gastric cancer	China; case-control	PB	306/ 615	enzymatic biochemical/ Plasma	M/F	60.6	Quartile >13.6 *vs* ≤8.0	1.82 (1.20-2.75)	Adjusted for age, sex, smoking status, and alcohol consumption.	7
Weinstein et al. (2008) [20]	colorectal cancer	Finland; Nested case-control	PB	275/ 275	HPLC /Serum	M	Colon:59.0 Rectal:58.0	Quintile Q5 *vs* Q1 (18.8*µ*mol/L vs 9.2*µ*mol/L)	1.22 (0.68-2.17)	Adjusted for age at randomization, body mass index, occupational and leisure physical activity, and intakes of vitamin D and iron.	8
Marchand et al. (2009) [25]	colorectal cancer	US; Nested case-control	PB	224/ 411	HPLC /Plasma	M/F	70.5	Quartile >11.8 *vs* ≤7.65	1.00 (0.56-1.79)	Adjusted for sex, ethnicity, study site, age at blood draw, fasting duration and date, time of blood draw, age at blood draw and hours of fasting prior to blood draw, hours of moderate or vigorous physical activity, processed meat, pack-years, BMI, ethanol, family history of colorectal cancer, history of colorectal cancer screening and plasma folate.	8
Miller et al. (2013) [18]	colorectal cancer	US; Nested case-control	PB	988/ 988	HPLC /Plasma	F	67	Quartile >9.85 *vs* ≤6.74	1.46 (1.05-2.04)	Adjusted for age, baseline BMI, ever had colonoscopy, smoking, physical activity (min/wk of moderate or strenuous activity), hormone replacement therapy, red blood cell folate, plasma vitamin B-12, and plasma pyridoxal-5#-phosphate.	8
F.-F. Chiang et al. (2014) [26]	colorectal cancer	China; case-control	HB	168/ 188	HPLC /Plasma	M/F	60.8	Higher *vs* lower	1.29 (1.14-1.47)	Adjusted for age, gender, body mass index, systolic blood pressure, creatinine, high sensitivity C-reactive protein, serum total cholesterol, smoking and drinking habits and B-vitamin supplement uses.	7
Gylling et al. (2014) [27]	colorectal cancer	Sweden; Nested case-control	PB	331/ 662	GC-MS /Plasma	M/F	59.7	Highest *vs* lowest	1.01 (0.72-1.43)	Adjusted for BMI, current smoking, recreational and occupational physical activity, and alcohol intake.	8
Fanidi et al. (2014) [28]	esophagus cancer	EPIC; Nested case-control	PB	74/ 944	HPLC /Plasma	M/F	62(all cases, including head, neck and esophagus)	Quartile Q4 *vs* Q1	1.19 (0.51-2.77)	Adjusting for country, sex, age at recruitment (in 5-year groups), educational attainment (in four groups), smoking status (never/former/current/missing), cotinine (quartiles defined among current smokers) and alcohol intake at recruitment (g/day).	8
Chang et al. (2015) [29]	gastric Cancer	China; case-control	PB	200/ 409	Chemiluminescent immunoassay/ Plasma	M/F	62.8	Quartile >13.1 *vs *≤6.7	1.12 (0.64-1.96)	Adjusted for age, gender, BMI, education, smoking pack-years, alcohol drinking frequency, H. pylori infection (in stomach cancer analyses), hepatitis B virus surface antigen (in liver cancer analyses), and plasma aflatoxin B1 levels (in liver cancer analyses); further adjusted for the other two plasma micronutrients in quintile distribution. (folate, vitamin B12)	7
Chang et al. (2015) [29]	esophagus cancer	China; case-control	PB	206/ 409	Chemiluminescent immunoassay/ Plasma	M/F	60.5	Quartile >13.1 *vs* ≤6.7	1.54 (0.87-2.72)	Adjusted for age, gender, BMI, education, smoking pack-years, alcohol drinking frequency, H. pylori infection (in stomach cancer analyses), hepatitis B virus surface antigen (in liver cancer analyses), and plasma aflatoxin B1 levels (in liver cancer analyses); further adjusted for the other two plasma micronutrients in quintile distribution. (folate, vitamin B12)	7
Miranti et al. (2017) [30]	non-cardia gastric adenocarcinoma	Finland; Nested case-control	PB	127/ 326	HPLC /Serum	M	58	Quartile >15.6 *vs* <11.0	1.16 (0.63-2.13)	Adjusted for age, BMI, number of cigarettes smoked, years of cigarette smoking, educational attainment, alcohol consumption, energy intake, fruits and vegetables.	8
Miranti et al. (2017) [30]	esophagogastric junctional adenocarcinoma	Finland; Nested case-control	PB	46/ 326	HPLC /Serum	M	59	Quartile >15.6 *vs* <11.0	0.85 (0.30-2.44)	Adjusted for age, BMI, number of cigarettes smoked, years of cigarette smoking, educational attainment, alcohol consumption, energy intake, fruits and vegetables.	8
Miranti et al. (2017) [30]	esophageal squamous cell carcinoma	Finland; Nested case-control	PB	60/ 326	HPLC /Serum	M	57	Quartile >15.6 *vs* <11.0	1.35 (0.56-3.33)	Adjusted for age, BMI, number of cigarettes smoked, years of cigarette smoking, educational attainment, alcohol consumption, energy intake, fruits and vegetables.	8

Abbreviations: EPIC: the European Prospective Investigation into Cancer and Nutrition; *vs*: versus; M: male; F: female; PB: population-based; HB: hospital-based; HPLC: High Performance Liquid Chromatography; GC-MS: gas chromatography-mass spectrometry; FPIA: fluorescence polarization immunoassay.

**Table 2:** Stratified analyses of homocysteine and digestive cancer risk.

Group	No. of comparisons	Summary OR (95% CI)	*P*	*I* ^*2*^ (%)
Total	16	1.27(1.15-1.39)	0.798	0.0
**Design**				
Nested case-control	11	1.16(0.99-1.37)	0.851	0.0
Case-control	5	1.33(1.18-1.49)	0.548	0.0
**Location **				
Asia	4	1.33(1.18-1.49)	0.386	1.2
America	3	1.38(1.05-1.80)	0.440	0.0
Europe	9	1.06(0.87-1.30)	0.990	0.0
**Cancer type**				
Esophagus cancer	4	1.31(0.89-1.93)	0.797	0.0
Gastric cancer	4	1.27(0.98-1.64)	0.152	43.3
Colorectal cancer	8	1.27(1.14-1.41)	0.780	0.0
**Source of control**				
PB	14	1.24(1.08-1.43)	0.682	0.0
HB	2	1.29(1.14-1.47)	0.857	0.0
**Material**				
Plasma	11	1.27(1.15-1.40)	0.533	0.0
Serum	5	1.26(0.91-1.74)	0.853	0.0
**Method of measurement**				
HPLC	9	1.29(1.16-1.44)	0.953	0.0
GC-MS	2	0.96(0.73-1.28)	0.634	0.0
FPIA	2	1.15(0.66-2.00)	0.758	0.0
Other method	3	1.53(1.15-1.39)	0.393	0.0
**Control definition**				
Healthy population	5	1.28(1.14-1.44)	0.850	0.0
Population free from cancer	11	1.25(1.07-1.47)	0.431	1.1

Abbreviations: PB: population-based; HB: hospital-based; HPLC: High Performance Liquid Chromatography; GC-MS: gas chromatography-mass spectrometry; FPIA: fluorescence polarization immunoassay.

## Data Availability

The data used to support the findings of our study have been deposited in PubMed. All 13 articles included in our meta-analysis can be found in PubMed; the PMID are 10206314, 12730865, 16638790, 18006931, 17438114, 18990766, 19661077, 19661077, 24280101, 25063522, 24975698, 25607998, and 28568053, respectively.
